# Supplementary material for: Determinants of Clinical Decision Making under Uncertainty in Dentistry: A Scoping Review
Source: Diagnostics (Basel). 2023 Mar 13;13(6):1076. doi: 10.3390/diagnostics13061076 (PMC10047498; doi:10.3390/diagnostics13061076)
Supplement: Supplementary file 1 [file diagnostics-13-01076-s001.zip › diagnostics-2117492-supplementary.pdf]

Supplementary Table S1

Extraction table consisting of the 64 articles that satisfied the inclusion criteria as identified by the PRISMA flowchart.

Study	Country	Study Design	Participants	Data Collection	Topic
Meyer BD, 2022 [22]	USA	Cross-sectional	Dental Specialists (Paediatric)	Questionnaire	Heuristics & Biases Related to the Decision-Making Process
Helayl Al Waqdani N, 2021 [25]	Saudi Arabia	Retrospective Cohort Study	Training Dental Specialists (Various)	Case histories of patients (personal, dental, medical), results of clinical examination, pre/post treatment PA, pulp status, peri-radicular status, diagnosis and treatment plan recorded	Heuristics & Biases Related to the Decision-Making Process
Careddu R, 2021 [27]	Ireland, Italy	Cross-Sectional	Dentists, Dental Specialists (Endodontic)	Questionnaire	Heuristics & Biases Related to the Decision-Making Process
Brondani MA, 2017 [28]	Canada	Cross-sectional	Patients	Semi-structured interview	Heuristics & Biases Related to the Decision-Making Process
Khatami S, 2012 [31]	USA	Cross-sectional	Dental Students	Hypothetical clinical scenario	Heuristics & Biases Related to the Decision-Making Process
Devlin H, 2012 [32]	UK	Cross-sectional	Dental Students	Questionnaire	Heuristics & Biases Related to the Decision-Making Process
Maupomé G, 2000 [33]	Canada	Qualitative	Dental Students	SPM consultation session and its review	Heuristics & Biases Related to the Decision-Making Process
Su H, 2014 [30]	USA	Cross-sectional	Dental Specialist	Questionnaire	Heuristics & Biases Related to the Decision-Making Process, Use of Existing Guidelines in Clinical Decision-Making
Luz LB, 2022 [35]	Brazil	Prospective Cohort Study	Dental Specialists (Endodontic), Dentists	Questionnaire	Clinical Factors Affecting Clinical Decision-Making
McGeown D, 2022 [23]	Ireland	Qualitative study	Dentists	Semi-structured interview	Clinical Factors Affecting Clinical Decision-Making
Kafantaris SN, 2020 [37]	Greece	Retrospective Cohort Study	Dental Specialists (Orthodontic, Prosthodontic, Oral Surgery)	Radiographs, diagnostic casts, clinical photographs, consultation with specialists	Clinical Factors Affecting Clinical Decision-Making
Evrard A, 2019 [38]	Belgium	Cross-sectional	Dental Specialists (Orthodontic)	Questionnaire	Clinical Factors Affecting Clinical Decision-Making
Leal SC, 2019 [39]	Brazil	Cross-Sectional	Dental Specialists (Paediatric)	Questionnaire	Clinical Factors Affecting Clinical Decision-Making
Cosyn J, 2012 [40]	Belgium	Descriptive, Cross-sectional	Dentists	Questionnaire	Clinical Factors Affecting Clinical Decision-Making
Fu PS, 2012 [41]	Taiwan	Case Report	Patient	Case report of a single teenager patient	Clinical Factors Affecting Clinical Decision-Making
Diniz MB, 2011 [42]	Brazil, USA	Cohort study (Prospective)	Dentists	Prescribed ICDAS, R scores and Treatment Decisions based on Clinical Photographs and Bitewings	Clinical Factors Affecting Clinical Decision-Making
Brocklehurst PR, 2010 [43]	UK	Descriptive, Cross-sectional	Dentists	Questionnaire	Clinical Factors Affecting Clinical Decision-Making

Moreira CH, 2007 [50]	Brazil	Qualitative	Dentists	Semi-structured interviews	Clinical Factors Affecting Clinical Decision-Making
Holmes RD, 2005 [46]	UK	Cohort Study	Patients (children)	Questionnaire	Clinical Factors Affecting Clinical Decision-Making
Liew J, 2021 [36]	UK	Cross-sectional	Training Dental Specialists (endodontics, prosthodontics, periodontics, oral surgery and implantology)	Questionnaire	Clinical Factors Affecting Clinical Decision-Making, Clinical Experience Affecting Confidence in Decision-Making
Al-Baghdadi M, 2019 [56]	UK	Qualitiative Descriptive	Dentists, Dental Specialists (OMFS)	Semi-structured interview	Clinical Factors Affecting Clinical Decision-Making, Clinical Experience Affecting Confidence in Decision-Making
Korduner EK, 2016 [57]	Sweden	Qualitative	Dentists	Semi-structured interview	Clinical Factors Affecting Clinical Decision-Making, Clinical Experience Affecting Confidence in Decision-Making
Ilgunas A, 2021 [24]	Sweden	Qualitative, inductive study	Dentists	Semi-structured interview	Clinical Factors Affecting Clinical Decision-Making, Heuristics & Biases Related to the Decision-Making Process
Mecler N, 2022 [49]	Brazil	Cross-sectional	Dental Students, Dentists, Dental Specialists	Questionnaire	Clinical Experience Affecting Confidence in Decision-Making
Swigart DJ, 2020 [54]	USA	Qualitative Descriptive Study	Dental Hygienists	Semi-structured interview	Clinical Experience Affecting Confidence in Decision-Making
Tolentino PHMP, 2019 [50]	Brazil	Cross-sectional	Dentists, Dental Specialists (Periodontic)	Questionnaire	Clinical Experience Affecting Confidence in Decision-Making
Keys T, 2019 [55]	Australia	Cross-sectional	Dentists	Questionnaire	Clinical Experience Affecting Confidence in Decision-Making
Bishti S, 2018 [51]	Germany	Cross-sectional	Dental Specialists (Prosthodontic)	Questionaire	Clinical Experience Affecting Confidence in Decision-Making
Williams KB, 2014 [53]	USA	Cross-sectional	Dental Students	Questionnaire	Clinical Experience Affecting Confidence in Decision-Making
Maidment Y, 2010 [58]	UK	Descriptive, Cross-sectional	Dentists	Questionnaire	Clinical Experience Affecting Confidence in Decision-Making
Klomp HJ, 2009 [48]	Germany	Descriptive, Cross-sectional	Dental Students, Dentists	Questionnaire	Clinical Experience Affecting Confidence in Decision-Making
Yusof ZY, 2008 [59]	Malaysia	Descriptive, Cross-sectional	Dentists	Questionnaire	Clinical Experience Affecting Confidence in Decision-Making
Cosyn J, 2007 [52]	Belgium	Prospective cohort	Dentists	A study form completed for every extraction performed over an 8-week period.	Clinical Experience Affecting Confidence in Decision-Making
Abbas B, 2022 [81]	Pakistan	Cross-sectional	Dentists, Patients	Questionnaire	Clinical Experience Affecting Confidence in Decision-Making, Use of Existing Guidelines in Clinical Decision-Making

Barber A, 2016 [60]	UK	Cross-sectional	Dental Students	Questionnaire	Patient Preferences & Perceptions in Clinical Decision-Making
Vernazza CR, 2015 [29]	UK	Qualitative	Dentists	Semi-structured interviews	Patient Preferences & Perceptions in Clinical Decision-Making
Azarpazhooh A, 2014 [61]	Canada	Cross-sectional	Patients	Questionnaire	Patient Preferences & Perceptions in Clinical Decision-Making
Ozhayat EB, 2010 [62]	Denmark	Cross-Sectional	Patients	Interview + Questionnaire	Patient Preferences & Perceptions in Clinical Decision-Making
Ozhayat EB, 2009 [63]	Denmark	Cross-Sectional	Patients	Interview + Questionnaire	Patient Preferences & Perceptions in Clinical Decision-Making
Gilmore D, 2006 [64]	UK	Cross-Sectional	Patients	Questionnaire	Patient Preferences & Perceptions in Clinical Decision-Making
Johnson BR, 2006 [17]	USA	RCT	Patients	Questionnaire	Patient Preferences & Perceptions in Clinical Decision-Making
Schouten BC, 2004 [65]	Netherlands	Qualitative	Patients	Questionnaire	Patient Preferences & Perceptions in Clinical Decision-Making
Watted N, 2000 [66]	Germany	Case Report	Patient	Case report of an orthodontic patient	Patient Preferences & Perceptions in Clinical Decision-Making
Dawson VS, 2021 [26]	Sweden	Qualitative	Dentists	Semi-structured interview	Patient Preferences & Perceptions in Clinical Decision-Making, Heuristics & Biases Related to the Decision-Making Process
Redford M, 1997 [34]	USA	Qualitative	Dentists, Patients	Semi-structured interviews	Patient Preferences & Perceptions in Clinical Decision-Making, Heuristics & Biases Related to the Decision-Making Process
Li S, 2022 [70]	USA	Retrospective Cross-sectional	Patients, Dentists, Medical Providers	Manual retrieval of pertinent information from 240 medical consults by dentists	Artificial Intelligence & Informatics in Decision-Making
Choi E, 2022 [18]	South Korea	Diagnostic Study	Patients, Dental Specialists (OMFS)	AI model output	Artificial Intelligence & Informatics in Decision-Making
Ehtesham H, 2020 [19]	Iran	Cross-sectional	Dental Specialist (Oral Medicine), Health information management experts	Questionnaire	Artificial Intelligence & Informatics in Decision-Making
Perakis N, 2019 [71]	Italy	Case Study	Dentists, Dental Specialists (Orthodontic)	Clinical photograph, digital wax-up, traditional wax up, cast	Artificial Intelligence & Informatics in Decision-Making
Tuzoff DV, 2019 [68]	Russia	Diagnostic Study	Dental Specialists (Dentomaxillofacial Radiology)	AI model output	Artificial Intelligence & Informatics in Decision-Making
Nam Y, 2018 [69]	South Korea	Cohort study	Patients	Retreived electronic dental records	Artificial Intelligence & Informatics in Decision-Making
Thanathornwong B, 2018 [16]	Thailand	Diagnostic Study	Patients (records), Dental Specialists	Retreived electronic dental records	Artificial Intelligence & Informatics in Decision-Making
Deshpande S, 2017 [72]	India	Cohort study	Dental Specialists (Prosthodontic)	Questionaire	Artificial Intelligence & Informatics in Decision-Making
White JM, 2011 [73]	USA	Cohort study (Restrospective)	Dentists, Dental Specialists (Paediatric)	Data collection from clinic EHR	Artificial Intelligence & Informatics in Decision-Making

Ríos Santos JV, 2008 [74]	Spain	Descriptive, Cross-sectional	Dental Students, Dentists	Questionnaire + Clinical Case Questions	Artificial Intelligence & Informatics in Decision-Making
Deniz N, 2022 [14]	Cyprus, Slovenia, South Korea, and Turkey	Case study	Qualifed Academicians	Delphi method, Smart pairwise comparisons, Borda voting and Simple Additive Weighting	Use of Existing Guidelines in Clinical Decision-Making
Amadi JU, 2021 [80]	Italy	Case report	Dentists, Dental Specialists (OMFS)	Clinical photographs, lateral cephalograms	Use of Existing Guidelines in Clinical Decision-Making
Tarnow DP, 2021 [77]	USA	Case report	Dental Specialist (Prosthodontic)	Patient history, diagnosis, treatment plan, clinical photographs	Use of Existing Guidelines in Clinical Decision-Making
Eliyas S, 2020 [78]	UK	Case report	Dentists	Patient history, diagnosis, treatment plan, clinical photographs	Use of Existing Guidelines in Clinical Decision-Making
Ehtesham H, 2019 [21]	Iran	Cross-sectional	Dentists, Dental Specialist (Oral Medicine)	National Delphi survey, usability evaluations questionnaire	Use of Existing Guidelines in Clinical Decision-Making
Brescia G, 2019 [79]	Italy	Case series	Dental Specialists (OMFS), Otolaryngologists	Symptoms, rigid nasal endoscopy, radiographs	Use of Existing Guidelines in Clinical Decision-Making
Chatzopoulos GS, 2018 [76]	USA	Cross-sectional	Patients	Retrieved electronic dental records	Use of Existing Guidelines in Clinical Decision-Making
Hänsel Petersson G, 2016 [82]	Sweden	Cross-sectional	Dentists, Patients	Questionnaire, history & clinical examination	Use of Existing Guidelines in Clinical Decision-Making
Korsch M, 2021 [75]	Germany	Cross-Sectional	Dental Specialists (OMFS)	Questionnaire	Use of Existing Guidelines in Clinical Decision-Making, Heuristics & Biases Related to the Decision-Making Process
